# Patterns of Co-occurring Comorbidities in People Living With HIV

**DOI:** 10.1093/ofid/ofy272

**Published:** 2018-10-24

**Authors:** Davide De Francesco, Sebastiaan O Verboeket, Jonathan Underwood, Emmanouil Bagkeris, Ferdinand W Wit, Patrick W G Mallon, Alan Winston, Peter Reiss, Caroline A Sabin, Pharmacokinetic and Clinical Observations in PeoPle Over fiftY (POPPY) study and the AGEhIV Cohort Study, Daphne Babalis, Marta Boffito, Laura Burgess, Paddy Mallon, Frank Post, Caroline A Sabin, Memory Sachikonye, Alan Winston, Jane Anderson, David Asboe, Marta Boffito, Lucy Garvey, Paddy Mallon, Frank Post, Anton Pozniak, Caroline A Sabin, Memory Sachikonye, Jaime Vera, Ian Williams, Alan Winston, Amanda Clarke, Jaime Vera, Andrew Bexley, Celia Richardson, Sarah Kirk, Rebecca Gleig, Marta Boffito, David Asboe, Anton Pozniak, Margherita Bracchi, Nicole Pagani, Maddalena Cerrone, Daniel Bradshaw, Francesca Ferretti, Chris Higgs, Elisha Seah, Stephen Fletcher, Michelle Anthonipillai, Ashley Moyes, Katie Deats, Irtiza Syed, Clive Matthews, Peter Fernando, Chido Chiwome, Shane Hardwick, Jane Anderson, Sifiso Mguni, Rebecca Clark, Rhiannon Nevin-Dolan, Sambasivarao Pelluri, Frank Post, Lucy Campbell, Selin Yurdakul, Sara Okumu, Louise Pollard, Beatriz Santana-Suarez, Paddy Mallon, Alan Macken, Bijan Ghavani-Kia, Joanne Maher, Maria Byrne, Ailbhe Flaherty, Sumesh Babu, Ian Williams, Damilola Otiko, Laura Phillips, Rosanna Laverick, Michelle Beynon, Anna-Lena Salz, Abigail Severn, Alan Winston, Lucy Garvey, Jonathan Underwood, Lavender Tembo, Matthew Stott, Linda McDonald, Felix Dransfield, Andrew Whitehouse, Laura Burgess, Daphne Babalis, Margaret Johnson, Nnenna Ngwu, Nargis Hemat, Martin Jones, Anne Carroll, Sabine Kinloch, Mike Youle, Sara Madge, Caroline A Sabin, Davide De Francesco, Emmanouil Bagkeris, P Reiss, F W N M Wit, M van der Valk, J Schouten, K W Kooij, R A van Zoest, E Verheij, S O Verboeket, B C Elsenga, M Prins, M F Schim van der Loeff, L del Grande, V Olthof, M Dijkstra, S Zaheri, M M J Hillebregt, Y M C Ruijs, D P Benschop, A el Berkaoui, N A Kootstra, A M Harskamp-Holwerda, I Maurer, M M Mangas Ruiz, A F Girigorie, B Boeser-Nunnink, W Zikkenheiner, F R Janssen, S E Geerlings, A Goorhuis, J W R Hovius, F J B Nellen, T van der Poll, J M Prins, P Reiss, M van der Valk, W J Wiersinga, M van Vugt, G de Bree, F W N M Wit, J van Eden, A M H van Hes, F J J Pijnappel, A Weijsenfeld, S Smalhout, M van Duinen, A Hazenberg, P G Postema, P H L T Bisschop, M J M Serlie, P Lips, E Dekker, N van der Velde, J M R Willemsen, L Vogt, J Schouten, P Portegies, B A Schmand, G J Geurtsen, F D Verbraak, N Demirkaya, I Visser, A Schadé, P T Nieuwkerk, N Langebeek, R P van Steenwijk, E Dijkers, C B L M Majoie, M W A Caan, H W van Lunsen, M A F Nievaard, B J H van den Born, E S G Stroes, W M C Mulder, S van Oorspronk

**Affiliations:** 1 Institute for Global Health, University College London, London, UK; 2 Department of Global Health, Amsterdam University Medical Centers, University of Amsterdam and Amsterdam Institute for Global Health and Development, Amsterdam, the Netherlands; 3 Division of Infectious Diseases, Imperial College London, London, UK; 4 UCD School of Medicine, Dublin, Ireland

**Keywords:** comorbidities, HIV, multimorbidity, patterns of comorbidities

## Abstract

**Background:**

The aims of this study were to identify common patterns of comorbidities observed in people living with HIV (PLWH), using a data-driven approach, and evaluate associations between patterns identified.

**Methods:**

A wide range of comorbidities were assessed in PLWH participating in 2 independent cohorts (POPPY: UK/Ireland; AGE_h_IV: Netherlands). The presence/absence of each comorbidity was determined using a mix of self-reported medical history, concomitant medications, health care resource use, and laboratory parameters. Principal component analysis (PCA) based on Somers’ *D* statistic was applied to identify patterns of comorbidities.

**Results:**

PCA identified 6 patterns among the 1073 POPPY PLWH (85.2% male; median age [interquartile range {IQR}], 52 [47–59] years): cardiovascular diseases (CVDs), sexually transmitted diseases (STDs), mental health problems, cancers, metabolic disorders, chest/other infections. The CVDs pattern was positively associated with cancer (*r* = .32), metabolic disorder (*r* = .38), mental health (*r* = .16), and chest/other infection (*r* = .17) patterns (all *P* < .001). The mental health pattern was correlated with all the other patterns (in particular cancers: *r* = .20; chest/other infections: *r* = .27; both *P* < .001). In the 598 AGE_h_IV PLWH (87.6% male; median age [IQR], 53 [48–59] years), 6 patterns were identified: CVDs, chest/liver, HIV/AIDS events, mental health/neurological problems, STDs, and general health. The general health pattern was correlated with all the other patterns (in particular CVDs: *r* = .14; chest/liver: *r* = .15; HIV/AIDS events: *r* = .31; all *P* < .001), except STDs (*r* = –.02; *P* = .64).

**Conclusions:**

Comorbidities in PLWH tend to occur in nonrandom patterns, reflecting known pathological mechanisms and shared risk factors, but also suggesting potential previously unknown mechanisms. Their identification may assist in adequately addressing the pathophysiology of increasingly prevalent multimorbidity in PLWH.

Widespread access to combination antiretroviral therapy (cART) has led to a marked improvement in the survival and life expectancy of people living with HIV (PLWH) [1, 2] and to an increase of proportions of PLWH over the age of 50 years in most settings [3]. As a result of this demographic shift, a high prevalence of multimorbidity, defined as the occurrence of multiple comorbidities within the same individual, has been reported among PLWH with implications for health outcomes and functional status [4–6]. However, knowledge about how individual comorbidities distribute or co-occur in the same individual among PLWH remains limited.

Managing individuals with multiple acute or chronic comorbidities is typically more challenging than managing individuals with a single condition [7, 8]. Current HIV guidelines [9, 10] recommend close monitoring of cardiovascular, metabolic, liver, kidney, and bone health and regular assessment of drug–drug interactions. Investigating common patterns, associations, interactions, and possible synergies between comorbidities could support further development of targeted interventions and guidelines for prevention and management of PLWH experiencing multiple comorbidities.

Assessment of patterns of comorbidities is complicated by “coincidental comorbidity”—the co-occurrence of 2 or more comorbidities by chance [11]. The identification of comorbidities that are more likely to occur together than would be expected by chance can reveal disease–disease interactions or indicate shared etiologies between comorbidities. It is therefore important to separate coincident (random) comorbidity from nonrandom comorbidity. Modern statistical methods allow the exploration of the underlying structure in the distribution of comorbidities, giving an overall picture of the broad pattern of how comorbidities cluster in a particular population.

A recent report from the HIV and Aging Working Group called for the collection of better observational data to identify common clusters (patterns) of comorbidities among PLWH and their impact on treatment and disease outcomes [12]. Although some studies have attempted to do so in the general population using statistical approaches without any a priori hypothesis [13], to the best of our knowledge, only 2 studies have focused on PLWH [14, 15]. These studies considered up to 15 comorbidities but were unable to include the broad spectrum of comorbidities and medical conditions that are commonly reported by PLWH, and therefore the patterns among a more comprehensive set of comorbidities observed in PLWH remain unclear.

The aims of this study were (1) to explore (noncoincidental) associations between comorbidities in 2 independent cohorts of PLWH, (2) to investigate common patterns of comorbidities using a data-driven statistical approach, and (3) to evaluate associations between the patterns identified.

## METHODS

This study is based on data from 2 European cohorts of PLWH: the POPPY study in the UK/Ireland and the AGE_h_IV study in the Netherlands. Both studies aimed to investigate the effects of aging and comorbidities in PLWH and assessed a wide range of comorbidities, as detailed below.

### The POPPY Study

#### Study Participants

The POPPY study recruited 2 cohorts of PLWH: an “older” group of PLWH aged ≥50 years and a younger group of PLWH aged 18–50 years, as described previously [16]. Inclusion criteria were documented presence of HIV infection, white or black-African ethnicity, likely route of HIV acquisition via sexual exposure, and ability to comprehend the study information leaflet. The younger group of PLWH was frequency-matched on gender, ethnicity, sexual orientation, and location (in or out of London) to the older PLWH. In addition, the study recruited a group of HIV-negative individuals aged ≥50 years, who was not included in the present analysis. Participants were recruited from HIV outpatient clinics between April 2013 and January 2016. The study was approved by the UK National Research Ethics Service (NRES; Fulham, London, UK, number 12/LO/1409). All participants provided written informed consent.

#### Data Collection

At study entry, a full clinical history of participants was captured detailing comorbidities and clinical conditions but also medications and health care resources used over the previous year. This information was self-reported by the study participants through a structured interview with trained staff who, where possible, also reviewed hospital notes to validate the presence of comorbidities. Participants were asked whether they ever experienced any of the comorbidities or medical conditions on a detailed list (Supplementary Table 1). For each organ system/pathophysiological group, participants were also asked to report a history of any other relevant comorbidity not included in the study protocol. Answers to these free-text questions were examined to update the existing list of comorbidities or to include additional ones. Reasons for any health care utilization over the previous year (including general practitioner visit, hospital visit, use of ambulance or hospital transport, psychiatrist/psychologist visit, nurse visit, specialist visit, hospital procedure, and health care provider) and information regarding any other (nonantiretroviral) medication received in the previous year were also scrutinized, and the presence of each comorbidity was updated accordingly. Congenital diseases and conditions with a prevalence <1.5% in the study population were subsequently excluded, yielding a total of 65 individual comorbidities from 19 organ system/pathogenic groups (Table 1).

**Table 1.** List of Comorbidities Considered by Organ System/Photogenic Group in the POPPY Study With Prevalence in the All POPPY PLWH Sample (n = 1073)

Organ System/ Pathogenic Group	Comorbidities	Prevalence, No. (%)	Collected in AGE_h_IV	Organ System/ Pathogenic Group	Comorbidities	Prevalence, No. (%)	Collected in AGE_h_IV
AIDS events	Tuberculosis	83 (7.7)	✓	Cardiovascular diseases	Myocardial infarction	41 (3.8)	✓
	Cytomegalovirus	28 (2.6)			Angina pectoris	34 (3.2)	✓
	Pneumocystis pneumonia	94 (8.8)	✓		Peripheral vascular disease	19 (1.7)	✓
	Kaposi’s sarcoma	70 (6.5)	✓		Hypertension	229 (21.3)	✓
	Other AIDS events	124 (11.6)	✓		Transient ischemic attack	31 (2.9)	✓
Infections	Varicella zoster virus	155 (14.4)	✓		Coronary artery bypass grafting	24 (2.2)	
	Fungal infection	54 (5.0)			Heart failure	25 (2.3)	✓
Endocrine diseases	Type 2 diabetes	62 (5.8)	✓	Bones and joint disorders	Joint inflammation/arthritis	224 (20.9)	✓
	Lipodystrophy/lipoatrophy	24 (2.2)	✓		Joint replacement	24 (2.2)	
	Dyslipidemia	293 (27.3)	✓		Osteopenia/osteoporosis	69 (6.4)	✓
	Hypothyroidism	22 (2.1)			Joint/back pain	118 (11.0)	
Mental health problems	Depression	367 (34.2)	✓		Osteoporotic fracture	136 (12.7)	✓
	Anxiety	67 (6.2)		Skin disorders	Eczema/dermatitis	109 (10.2)	
	Panic attacks	20 (1.9)			Psoriasis	47 (4.4)	
	Sleeping problems	71 (6.6)	✓	Sexually transmitted diseases	Chlamydia	245 (22.8)	✓
Nervous system problems	Dizziness/vertigo	116 (10.8)	✓		Gonorrhea	457 (42.6)	✓
	Loss of consciousness	31 (2.9)	✓		Human papilloma virus	99 (9.2)	
	Epilepsy	45 (4.2)	✓		Herpes simplex virus	122 (11.4)	
	Encephalitis	16 (1.5)	✓		Lymphogranuloma venereum	46 (4.3)	
	Peripheral neuropathy	43 (4.0)	✓		Syphilis	326 (30.4)	✓
	Migraine/headache	33 (3.1)		Gastro-intestinal disorders	Hernia	27 (2.5)	
Respiratory diseases	Asthma/bronchitis/chronic obstructive pulmonary disease	264 (24.6)	✓		Gastro-esophageal reflux disease	70 (6.5)	
	Pneumonia	44 (4.1)	✓		Irritable bowel syndrome	30 (2.8)	
	Chest infection	114 (10.6)		Genitourinary disorders	Urinary incontinence	62 (5.8)	✓
	Hay fever/allergy	77 (7.2)	✓		Urinary tract infections	31 (2.9)	
Hepatitis	Hepatitis A	44 (4.1)			Erectile dysfunction	75 (7.0)	✓
	Hepatitis B	147 (13.7)	✓		Nonspecific urethritis	58 (5.4)	
	Hepatitis C	75 (7.0)	✓		Prostate dysfunction	31 (2.9)	
Renal problem	Renal problem	28 (2.6)	✓		Kidney stones	28 (2.6)	
Cancer	Skin cancer	67 (6.2)	✓	Ear dysfunction	Ear dysfunction	40 (3.7)	
	Hematological cancer	22 (2.1)	✓	Eye problem	Eye problem	81 (7.5)	✓
	Solid organ cancer	75 (7.0)	✓	Vitamin D deficiency	Vitamin D deficiency	19 (1.8)	✓
Anemia	Anemia	28 (2.6)	✓				

Abbreviation: PLWH, people living with HIV.

### The AGE_h_IV Study

#### Study Participants

The AGE_h_IV study recruited PLWH aged ≥45 years from the HIV outpatient clinic of the Academic Medical Centre in Amsterdam, the Netherlands [17], between October 2010 and September 2012. Inclusion criteria were age ≥45 years and laboratory-confirmed presence of HIV infection. Although a control group of HIV-negative individuals was also enrolled in the study, these individuals were not included in the present analysis. The study protocol was approved by the local ethics review committee (ClinicalTrial.gov identifier NCT01466582). All participants provided written informed consent.

#### Data Collection

Participants underwent standardized screening for several comorbidities, including a questionnaire concerning personal medical history, use of medications (both prescribed and over the counter), and participation in population screening programs. Information concerning blood pressure, anthropometrics, vascular elasticity, respiratory function (via spirometry test), cognitive function, frailty, and bone densitometry (using DXA scan) was also obtained, in addition to blood and urine samples for extensive laboratory testing and historical HIV characteristics (from the Dutch HIV Monitoring Foundation registry).

Information collected was used to derive the presence/absence of 42 of the 65 comorbidities in Table 1, as well as a further 4 (esophageal candidiasis, hyperparathyroidism, liver problems, and thrombocytopenia). Whenever possible, patient-reported comorbidities were validated using hospital records. The list of comorbidities obtained and the source of information for each comorbidity are reported in Supplementary Table 2.

### Statistical Analysis

Comparison of participants’ characteristics between the older POPPY PLWH and AGE_h_IV PLWH was performed using the chi-square or Wilcoxon rank-sum test, as appropriate. Pairwise associations between comorbidities were assessed using the Somers’ *D* statistic for binary variables, as proposed by Ng et al. [18], which has been shown to perform better than other measures of agreement in detecting nonrandom comorbidity [19]. Briefly, Somers’ *D* measures the degree of association between 2 comorbidities other than that given by chance alone (the product of the prevalence of the individual comorbidities). Somers’ *D* ranges from –1 when there is perfect disagreement between the 2 comorbidities (ie, all individuals have either 1 or the other comorbidity) to 1 when there is perfect agreement (ie, all individuals either have both comorbidities or neither comorbidity), with 0 indicating that agreement equals that given by chance alone. The significance of the Somers’ *D* statistic was evaluated using permutation tests, with *P* < .001 indicative of significant nonrandom association (reflecting the high number of pair-wise associations tested, ie, 2080).

Principal component analysis (PCA) was applied to the matrix containing the pairwise associations (as measured by Somers’ *D*) between the comorbidities. PCA is a data reduction method that transforms the original set of variables into a smaller set of principal components (PCs), which are linear combinations of the original variables. These PCs are determined so that they retain as much of the variability in the data set as possible, with the first component retaining the greatest amount of the variation present and the other components progressively retaining a decreasing amount of variation [20]. PCs can be interpreted as patterns of comorbidities, ie, groups of comorbidities frequently associated with each other. A comorbidity was regarded to be associated with a pattern if its correlation with the pattern was >.40. We adopted an *oblimin* rotation to allow PCs (patterns) to be associated within each other, thereby allowing the possibility of multiple patterns being present in the same individual. The number of PCs to be extracted was determined using the scree plot and the very simple structure criterion [21].

For each participant and each pattern, a severity score for that pattern was obtained using data on the presence/absence of comorbidities and coefficients returned by the PCA. Correlations between patterns’ severity scores were evaluated using Spearman’s rank correlation coefficient (*r*). All the analyses were performed separately in all POPPY PLWH, in older POPPY PLWH only, and in AGE_h_IV PLWH using the statistical software R, version 3.3.3.

## RESULTS

### Characteristics of the POPPY and AGE_h_IV Study Participants

The POPPY study recruited 699 older and 374 younger PLWH; 598 PLWH were recruited into the AGE_h_IV Cohort Study. Sociodemographic and HIV-related characteristics are summarized in Table 2. POPPY participants were predominantly male (85.2%), of white ethnicity (84.1%), and men who have sex with men (MSM; 76.0%). The median (interquartile range [IQR]) CD4^+^ T-cell count was 624 (475–811) cells/μL, and 89.9% had a suppressed viral load (<50 copies/mL).

**Table 2.** Characteristics of POPPY and AGE_h_ IV PLWH

	POPPY			AGE_h_IV
	All PLWH (n = 1073)	PLWH ≥50 (n = 699)	PLWH <50 (n = 374)	PLWH ≥45 (n = 598)
Gender, No. (%)				
Male	914 (85.2)	612 (87.5)	302 (80.8)	524 (87.6)
Female	159 (14.8)	87 (12.5)	72 (19.2)	74 (12.4)
Age, median (IQR), y	52 (47–59)	57 (53–62)	43 (37–47)	53 (48–59)
Ethnicity, No. (%)^a^				
Black-African	171 (15.9)	96 (13.7)	75 (19.8)	74 (12.4)
White	902 (84.1)	603 (86.3)	299 (80.2)	513 (85.8)
Other/unknown	0 (0.0)	0 (0.0)	0 (0.0)	11 (1.8)
Sexual orientation, No. (%)				
MSM/homosexual	816 (76.0)	548 (78.4)	268 (71.7)	369 (70.4)
Heterosexual	257 (24.0)	151 (21.6)	106 (28.3)	177 (29.6)
BMI, median (IQR), kg/m^2^	25.5 (23.2–28.2)	25.7 (23.4–28.5)	25.2 (23.0–27.8)	24.3 (22.4–26.7)
Duration of HIV, median (IQR), y	13.2 (7.8–20.5)	15.8 (9.8–22.4)	9.7 (5.5–15.2)	12.0 (6.6–17.0)
CD4^+^ T-cell count, median (IQR), cells/mm^3^	624 (476–811)	610 (468–792)	661 (500–847)	565 (433–740)
Nadir CD4^+^ count, median (IQR), cells/mm^3^	202 (101–304)	180 (85–273)	253 (152–376)	170 (70–260)
On ART, No. (%)	1046 (97.5)	690 (98.7)	356 (95.2)	573 (95.8)
HIV RNA <50 copies/mL, No. (%)	965 (89.9)	644 (91.8)	323 (86.4)	545 (91.6)

^a^For the AGE_h_IV participants, white refers to Dutch origin, black-African to African origin.

Abbreviations: ART, antiretroviral therapy; BMI, body mass index; IQR, interquartile range; MSM, men who have sex with men; PLWH, people living with HIV.

Similarly, the majority of PLWH recruited in the AGE_h_IV study were male (87.6%), of Dutch origin (85.8%), and MSM (70.4%). When compared with the older POPPY PLWH, they were younger (*P* < .001) and had a lower body mass index (BMI; *P* < .001); however, there were no significant differences in terms of gender (*P* = .91), ethnicity (*P* = .61), or sexual orientation (*P* = .09).

### Individual Comorbidities

In POPPY PLWH, the prevalence of comorbidities ranged from 1.6% (encephalitis) to 42.6% (gonorrhea) (Table 1), with also depression (34.2%), syphilis (30.4%), and dyslipidemia (27.3%) among the most prevalent comorbidities. In total, 97.2% of all POPPY PLWH and 98.6% of older POPPY PLWH reported ≥1 comorbidity with a median (IQR) of 5 (3–7) and 6 (3–8) comorbidities per individual, respectively. Of the comorbidities considered in AGE_h_IV PLWH, hypertension (43.1%), osteopenia/osteoporosis (42.6%), lipodystrophy/lipoatrophy (32.1%), and candidiasis (31.9%) were the most common (Supplementary Table 2). Overall, 98.7% had ≥1 comorbidity, with a median (IQR) of 5 (3–7) comorbidities per individual.

### Nonrandom Associations Between Comorbidities

**Figure 1.**
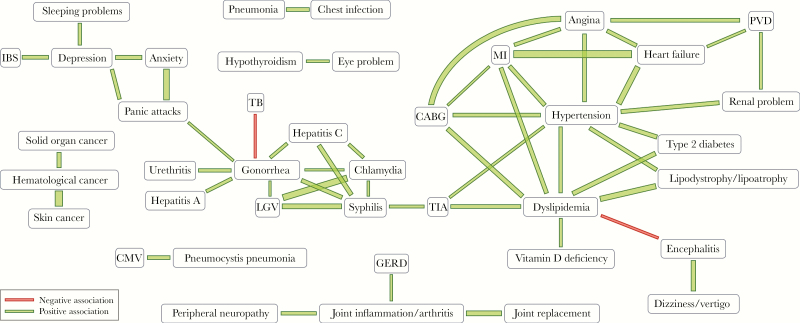
Significant nonrandom associations between comorbidities (as indicated by a significant Somers’ *D* at the 0.1% significance level) in all POPPY PLWH (n = 1073). The thickness of the line is directly proportional to the absolute value of the Somers’ D. Abbreviations: CABG, coronary artery bypass graft; CMV, cytomegalovirus; GERD, gastro-esophageal reflux disease; IBS, irritable bowel syndrome; LGV, lymphogranuloma venereum; MI, myocardial infarction; PLWH, people living with HIV; PVD, peripheral vascular disease; TB, tuberculosis; TIA, transient ischemic attack.

Significant nonrandom associations, based on the Somers’ *D* statistic, among POPPY PLWH are depicted in Figure 1 (all pairwise Somers’ *D* are reported in the Supplementary Data). Nonrandom associations within several cardiovascular diseases (CVDs) were significant (top-right part of Figure 1); these included hypertension, angina, heart failure, type 2 diabetes, lipodystrophy/lipoatrophy, dyslipidemia, transient ischemic attack (TIA), coronary artery bypass graft (CABG), myocardial infarction (MI), and renal problems. Other strong nonrandom associations were identified within mental health problems (depression, anxiety, and panic attacks), with depression also being associated with sleeping problems and irritable bowel syndrome (IBS), cancers (hematological cancer with both solid organ and skin cancer), and sexually transmitted diseases (STDs; in particular gonorrhea, chlamydia, lymphogranuloma venereum [LGV], syphilis, and hepatitis C virus).

**Figure 2.**
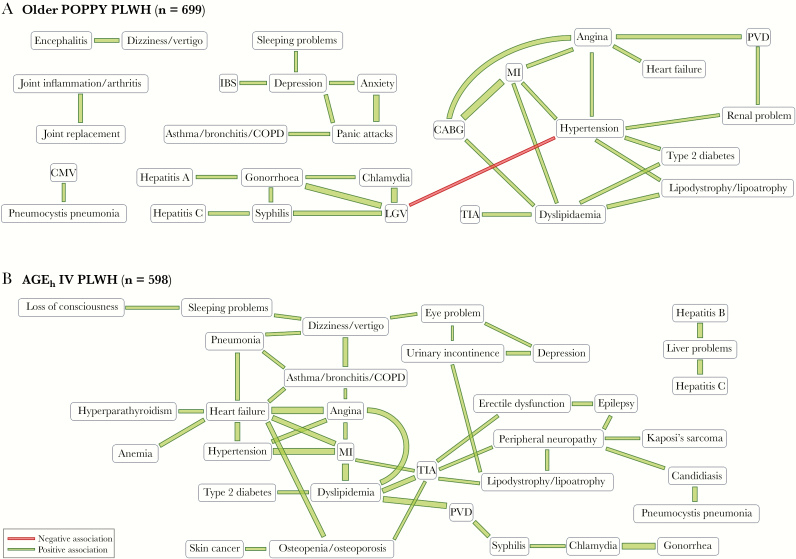
Significant nonrandom associations between comorbidities (significant Somers’ *D* at the 0.1% significance level) in all older POPPY PLWH (A) and AGE_h_IV PLWH (B). The thickness of the line is directly proportional to the absolute value of the Somers’ *D*. Abbreviations: CABG, coronary artery bypass graft; CMV, cytomegalovirus; COPD, chronic obstructive pulmonary disease; IBS, irritable bowel syndrome; LGV, lymphogranuloma venereum; MI, myocardial infarction; PLWH, people living with HIV; PVD, peripheral vascular disease; TIA, transient ischemic attack.

Most of these nonrandom associations were maintained when the analysis was restricted to older POPPY PLWH (Figure 2A). In addition, a strong nonrandom positive association was found between panic attacks and asthma/bronchitis/chronic obstructive pulmonary disease (COPD), and a significantly lower than expected co-occurrence of hypertension and LGV. A few of these significant nonrandom associations were also found in the AGE_h_IV PLWH (Figure 2B), namely the associations between MI, angina and hypertension, those of dyslipidemia with TIA, MI and type 2 diabetes, and those between gonorrhea and chlamydia and between angina and heart failure.

### Patterns of Comorbidities

The PCA in POPPY PLWH yielded 6 components explaining 24.4% of the total variation in the original 65 comorbidities. Correlations between comorbidities and each pattern are reported in the Supplementary Data, with Table 3 reporting only comorbidities with a correlation >0.4 with each pattern. The first PC accounted for 6.2% of the variance and encompassed CVDs such as angina, CABG, MI, heart failure, hypertension, peripheral vascular disease (PVD), and renal problems. The second PC, with strong correlations with STDs (gonorrhea, chlamydia, LGV, syphilis, and hepatitis C), accounted for 5.0% of the variability. The third PC strongly correlated with mental health problems (depression, anxiety, and panic attacks). The fourth and fifth PCs included cancers (hematological, skin, and solid organ cancer) and metabolic disorders (dyslipidemia, lipodystrophy/lipoatrophy, and hypertension), respectively. Finally, the sixth PC had the strongest correlation with chest problems (pneumonia, asthma/bronchitis/COPD, and chest infection), cytomegalovirus, and dizziness/vertigo.

**Table 3.** PC Extracted by the PCA in All POPPY PLWH (A, n = 1073), Older POPPY PLWH Only (B, n = 699), and AGE_h_IV PLWH (C, n = 598) Groups

PC (% of Variance Explained)	Meaning	Comorbidities With Correlation >0.4 (Correlation With the PC)
A, All POPPY PLWH (n = 1073)		
1 (6.2)	CVDs	Angina (0.66), CABG (0.66), MI (0.64), heart failure (0.59), hypertension (0.54), PVD (0.53), renal problem (0.42)
2 (4.9)	STDs	Gonorrhea (0.77), syphilis (0.67), LGV (0.66), chlamydia (0.64), hepatitis C (0.40)
3 (3.9)	Mental health	Depression (0.79), anxiety (0.58), panic attacks (0.50)
4 (3.5)	Cancers	Hematological cancer (0.75), skin cancer (0.64), solid organ cancer (0.49)
5 (3.1)	Metabolic	Dyslipidemia (0.71), lipodystrophy/lipoatrophy (0.57), hypertension (0.44)
6 (2.8)	Chest and other infections	CMV (0.49), pneumonia (0.49), dizziness/vertigo (0.44), asthma/bronchitis/COPD (0.42), chest infection (0.41)
B, Older POPPY PLWH (n = 699)		
1 (5.7)	CVDs	Angina (0.67), heart failure (0.65), PVD (0.62), CABG (0.58), MI (0.57), hypertension (0.54), renal problems (0.44)
2 (5.0)	STDs	Gonorrhea (0.74), LGV (0.72), syphilis (0.69), chlamydia (0.63), hepatitis C (0.46)
3 (4.1)	Mental health and asthma	Depression (0.79), anxiety (0.58), panic attacks (0.50), asthma/bronchitis/COPD (0.45), dizziness/vertigo (0.40)
4 (3.7)	Cancers	Hematological cancer (0.78), skin cancer (0.64), solid organ cancer (0.54)
5 (3.3)	Metabolic	Dyslipidemia (0.71), lipodystrophy/lipoatrophy (0.43)
6 (3.0)	Other	Hypothyroidism (0.46), other AIDS events (0.46)
C, AGE_h_IV PLWH (n = 598)		
1 (9.7)	CVDs	Dyslipidemia (0.73), Hypertension (0.64), PVD (0.60), angina pectoris (0.54), MI (0.51)
2 (6.2)	Chest/liver	Asthma/bronchitis/COPD (0.59), liver problems (0.55), hepatitis B (0.47), dizziness/vertigo (0.43)
3 (4.9)	HIV/AIDS events	Peripheral neuropathy (0.63), candidiasis (0.62), lipodystrophy (0.53), other AIDS event (0.48)
4 (4.9)	Mental health & neurological problems	Eye problem (0.64), depression (0.63), urinary incontinence (0.50), loss of consciousness (0.48), sleeping problems (0.42), dizziness/vertigo (0.40)
5 (4.4)	STDs	Chlamydia (0.70), gonorrhea (0.67), syphilis (0.62)
6 (4.3)	General health	Heart failure (0.88), hyperparathyroidism (0.54), vitamin D deficiency (0.48), anemia (0.45)

For each principal component, comorbidities with a high correlation (>0.4) are reported.

Abbreviations: CABG, coronary artery bypass graft; CMV, cytomegalovirus; COPD, chronic obstructive pulmonary disease; CVD, cardiovascular disease; LGV, lymphogranuloma venereum; MI, myocardial infarction; PC, principal component; PLWH, people living with HIV; PCA, principal component analysis; PVD, peripheral vascular disease; STD, sexually transmitted disease.

In the older POPPY PLWH, the PCA returned 6 components explaining 24.8% of the total variability (Table 3; Supplementary Data). Several components were similar to those found among all POPPY PLWH: CVDs (first PC), STDs (second PC), cancers (fourth PC), and metabolic disorders (fifth PC). Similar to the “mental health” pattern in all POPPY PLWH, the third PC included mental health problems but with the addition of asthma/bronchitis/COPD and dizziness/vertigo. Finally, the sixth PC correlated with hypothyroidism (0.46) and other AIDS events.

Some of the 6 patterns returned by the PCA in the AGE_h_IV PLWH, explaining 34.2% of the total variance, resembled those identified in the older POPPY PLWH. The first PC correlated with CVDs (dyslipidemia, hypertension, PVD, angina pectoris, and MI). The second PC included chest (asthma/bronchitis/COPD) and liver problems (with hepatitis B). The third and fourth PCs were related to HIV/AIDS events and mental health/neurological problems, respectively. The fifth included STDs (gonorrhea, chlamydia, and syphilis), and the sixth encompassed disorders associated with general health such as anemia, vitamin D deficiency, hyperparathyroidism, and heart failure.

### Correlations Between Patterns

Correlations between patterns’ severity scores are reported in Table 4. In all POPPY PLWH, positive correlations of severity scores in the CVDs pattern were strongest with scores in the cancer (*r* = .32) and metabolic patterns (*r* = .38) and moderate with those in the mental health (*r* = .16) and chest/other infection (*r* = .17) patterns (all *P* < .001). A significant negative correlation was also found between the CVDs and STDs patterns (*r* = –.10; *P* = .001), suggesting that PLWH with a higher burden of CVDs tend to have a lower number of STDs and vice versa. Generally, the severity of the mental health pattern was positively correlated with the severity of all other patterns, with the strongest evidence for cancers (*r* = .20; *P* < .001) and chest and other infections (*r* = .27; *P* < .001). Chest/other infections was also positively associated with cancers and metabolic patterns (*r* = .27 and *r* = .25, respectively; both *P* < .001).

**Table 4.** Correlation Between Patterns’ Severity Scores in All POPPY PLWH (A, n = 1073), Older POPPY PLWH Only (B, n = 699), and AGE_h_IV PLWH (C, n = 598) Groups

A, All POPPY PLWH (n = 1073)					
	STDs	Mental Health	Cancers	Metabolic	Chest & Other Infections
CVDs	–0.10 (*P* = .001)	0.16 (*P* < .001)	0.32 (*P* < .001)	0.38 (*P* < .001)	0.17 (*P* < .001)
STDs		0.11 (*P* < .001)	0.07 (*P* = .01)	0.05 (*P* = .13)	0.03 (*P* = .27)
Mental health			0.20 (*P* < .001)	0.16 (*P* < .001)	0.27 (*P* < .001)
Cancers				0.21 (*P* < .001)	0.27 (*P* < .001)
Metabolic					0.25 (*P* < .001)
B, Older POPPY PLWH (n = 699)					
	STDs	Mental Health & Asthma	Cancers	Metabolic	Other
CVDs	0.05 (*P* = .18)	0.22 (*P* < .001)	0.26 (*P* < .001)	0.34 (*P* < .001)	–0.04 (*P* = .24)
STDs		0.11 (*P* = .004)	0.07 (*P* = .06)	0.09 (*P* = .02)	0.08 (*P* = .03)
Mental health and asthma			0.18 (*P* < .001)	0.12 (*P* = .002)	0.25 (*P* < .001)
Cancers				0.11 (*P* = .005)	0.14 (*P* < .001)
Metabolic					–0.10 (*P* = .008)
C, AGE_h_IV PLWH (n = 598)					
	Chest/Liver	HIV/AIDS Events	Mental Health/Neurological Problems	STDs	General Health
CVDs	0.02 (*P* = .70)	0.18 (*P* < .001)	0.05 (*P* = .21)	–0.07 (*P* = .11)	0.14 (*P* < .001)
Chest/liver		0.21 (*P* < .001)	0.05 (*P* = .23)	–0.06 (*P* = .14)	0.15 (*P* <.001)
HIV/AIDS events			0.10 (*P* = .02)	0.04 (*P* = .30)	0.31 (*P* < .001)
Mental health/neurological problems				–0.01 (*P* = .90)	0.08 (*P* = .05)
STDs					–0.02 (*P* = .64)

Abbreviations: CVD, cardiovascular disease; PLWH, people living with HIV; STD, sexually transmitted disease.

In the older POPPY PLWH, higher CVDs severity scores were correlated with higher mental health/asthma (*r* = .22), cancer (*r* = .26), and metabolic (*r* = .34) scores (all *P* < .001). Mental health/asthma scores also correlated with all the remaining patterns, with correlations ranging between 0.11 (with STDs) and 0.25 (with other). Among AGE_h_IV PLWH, association of CVDs was strongest with HIV/AIDS events (*r* = .18; *P* < .001) and general health problems (*r* = .14; *P* < .001) but weak with other patterns. A higher severity of the general health pattern was correlated with higher severity in all the other patterns (CVDs; chest/liver: *r* = .15; *P* < .001; HIV/AIDS events: *r* = .31; *P* < .001; mental health/neurological problems: *r* = .08; *P* = .05) but not with STDs (*r* = –.02; *P* = .64).

## DISCUSSION

This study explored associations between a wide range of comorbidities and identified common patterns occurring in 2 independent cohorts of PLWH. Our findings suggest that, in PLWH, comorbidities do not co-occur at random and, in general, are likely to cluster in specific patterns, some of which are consistent across different cohorts. In particular, we found that patterns of CVDs, metabolic disorders, STDs, and mental health problems are present in treated PLWH from both the UK/Ireland and the Netherlands. Our study adds to the 2 previous studies reporting patterns of comorbidities identified through purely statistical approaches [14, 15] by considering a wider range of comorbidities and validating the results in 2 independent cohorts.

Nonrandom associations between CVDs such as angina, hypertension, MI, CABG, and heart failure (which formed 1 of the patterns identified) reflect previously known pathological mechanisms and were also previously reported in HIV-positive veterans [14] and in the general population [22–24]. Similarly, patterns of metabolic disorders are often reported in conjunction with CVDs in both PLWH [15] and the general population [22, 23]. On the other hand, contrary to other studies that used a similar data-driven approach in PLWH [14, 15] and in the general population [22–24], we found frequent co-occurrence of STDs, likely due to exposure to some shared risk factors (ie, risk-taking sexual behaviors) that are highly prevalent among populations of PLWH [25]. Moreover, in 1 of the 2 cohorts analyzed, we found links between several opportunistic infections (ie, candidiasis, pneumocystis pneumonia, and other AIDS-defining events). These links likely reflect past immunosuppression and were mainly present in long-term survivors, as also indicated by the association of the pattern’s severity score with age and time since HIV diagnosis (data not shown).

Although associations between mental health problems like depression, anxiety, and panic attacks can reflect true underlying psychological distress and co-occurrence patterns have been reported in other studies [23, 24], they may also highlight a monitoring bias. Individuals reporting 1 of the problems tend to be more likely to also report the others. Moreover, in our study, mental health disorders were associated not only with each other, but also with neurological problems, especially in older PLWH. These results were also reported by Kirchberger et al. [26] and are consistent with the growing evidence about the bidirectional link between mental health and neurological disorders [27].

Interestingly, there was some overlap between patterns, as suggested by significant correlations between patterns’ severity scores. In particular, mental health problems appeared to be associated with almost all other patterns, including those of CVDs, STDs, and metabolic disorders. Although these findings are in line with reports of the strong link between physical and mental health [28, 29], the nature of these associations is likely to be bidirectional. Poor physical health can lead to an increased risk of developing mental health problems, but, at the same time, individuals with several mental health disorders are often more likely to experience physical health conditions [30].

There are some limitations to our study that need to be considered. First, not all of the comorbidities considered required a medical diagnosis, and some consisted more of symptoms or treatments rather than actual diseases. Although no uniform list of comorbidities and medical conditions exists to define multimorbidity, the list of comorbidities considered here aimed to capture the broad spectrum of conditions affecting PLWH. Nevertheless, the use of a more standardized list of conditions and criteria for the ascertainment of the presence of conditions (eg, ICD codes) could have provided a more uniform set of conditions and more replicable results. Second, the self-reported nature of data collection may have led to under- or over-reporting of some comorbidities. Although the 2 cohort studies (POPPY and AGE_h_IV) were conducted following similar protocols, not all comorbidities were assessed by both studies and differences across studies exist in how the presence/absence of some comorbidities was defined, which may have resulted in the differences in the patterns identified. Moreover, both the POPPY and AGE_h_IV cohorts were designed to be representative of the population of PLWH seen in care in the respective countries, where the majority of PLWH are white MSM; therefore, results could be less generalizable to cohorts of PLWH that include larger proportions of women, people of black-African ethnicity, or to cohorts in different HIV epidemic settings.

Our findings could be useful for both research and clinical purposes. With an increasingly aging population of PLWH [3] and the consequent increase in the prevalence of multimorbidity and its associated health care costs [31], a better understanding of how comorbidities cluster together would enable us to develop targeted interventions and appropriate guidelines addressing the needs of PLWH with multiple comorbidities. Further studies may help to elucidate the possible pathophysiological pathways linking conditions with demonstrated co-occurrence prevalences higher than those expected by chance alone and their impact on health and treatment outcomes.

## Supplementary Data

Supplementary materials are available at *Open Forum Infectious Diseases* online. Consisting of data provided by the authors to benefit the reader, the posted materials are not copyedited and are the sole responsibility of the authors, so questions or comments should be addressed to the corresponding author.

Supplementary Tables
