# Supplementary material for: Patterns of Co-occurring Comorbidities in People Living With HIV
Source: Open Forum Infect Dis. 2018 Oct 24;5(11):ofy272. doi: 10.1093/ofid/ofy272 (PMC6239080; doi:10.1093/ofid/ofy272)
Supplement: Supplementary Tables [file ofy272_suppl_supplementary_tables.docx]

# **Supplementary tables**

**Supplementary Table 1:** list of comorbidities and medical conditions assessed via structured questionnaire in the POPPY study

| **Organ system/pathogenic group** | **Comorbidity** |
| --- | --- |
| AIDS defining events | Tuberculosis (TB) |
|  | Other AIDS events |
| Infections and paracytic | Hepatitis B |
|  | Hepatitis C |
|  | Other Infections |
| Sexually transmitted diseases (STDs) | Syphilis |
|  | Gonorrhoea |
|  | Chlamydia |
|  | Lymphogranuloma venereum (LGV) |
|  | Other STD |
| Endocrine disease | Type 1 Diabetes |
|  | Type 2 Diabetes |
|  | Thyroid disease |
|  | Other endocrine diseases |
| Blood diseases | Blood and blood forming organ events |
| Mental health | Depression |
|  | Diagnosed depression (treated by a doctor) |
|  | Other mental disorders |
| Nervous system | Parkinson's disease or any other movement disorder |
|  | Dizziness or vertigo |
|  | Loss of consciousness for 30 minutes or more |
|  | Brain surgery |
|  | Encephalitis |
|  | Epilepsy |
|  | Peripheral neuropathy |
|  | Other nervous system disorders |
| Cardiovascular disease | Myocardial infarction (MI) |
|  | Coronary artery bypass surgery or percutaneous transluminal coronary angioplasty (CABG/PCTA) |
|  | Cerebrovascular accident or transient ischemic attack (CVA/TIA) |
|  | Other cardiovascular disease |
|  | Heart Failure |
|  | Angina Pectoris |
|  | Narrowed blood vessels in legs or abdomen |
| Chest disease | Asthma, bronchitis, pulmonary emphysema or chronic obstructive pulmonary disease (COPD) |
|  | other chest disease |
| Gastro-intestinal | Persistent bowel disorder over last 3 months |
|  | Other liver disease |
|  | Other gastro-intestinal disorders |
| Renal/Urinary/Reproductive | End-stage renal disease (receipt of dialysis or  renal transplant) |
|  | Urinary incontinence requiring treatment |
|  | Other genitourinary disorders |
| Skin | Any skin disorders |
| Joint, bone and connective tissue | Joint inflammation or rheumatoid or osteoarthritis |
|  | Arthritis of knee or hip |
|  | Joint replacement |
|  | Fractures |
|  | Congenital bone disease (osteogenesis imperfecta) |
|  | Other joint, bone or connective tissue disorders |
| Congenital | Congenital disorders |
| Injury and poisoning | Injury or poisoning |
| Cancer | Any form of cancer (if not covered elsewhere) |
| Chronic disease | Any chronic diseases not recorded elsewhere |

**Supplementary Table 2:** List of comorbidities with source of information and prevalence in the AGE_h_IV study (n=598)

| **Organ system/**  **pathogenic group** | **Comorbidities** | **Source of information** | **Prevalence**  **n (%)** |
| --- | --- | --- | --- |
| AIDS events | Tuberculosis (TB) | Hospital records | 17 (2.8%) |
|  | Pneumocystis pneumonia | Hospital records | 53 (8.9%) |
|  | Kaposi’s sarcoma | Hospital records | 47 (7.8%) |
|  | Candidiasis | Hospital records | 191 (31.9%) |
|  | Other AIDS events | Hospital records | 159 (26.6%) |
| Infections | Varicella zoster virus (VZV) | Hospital/GP records | 122 (20.4%) |
| Endocrine diseases | Type 2 diabetes | Medical history \| HbA_1c_ ≥ 48 mmol/l \| blood glucose (≥11.1 non-fasting, ≥7 fasting) \| Medication | 37 (6.2%) |
|  | Lipodystrophy/Lipoatrophy | Hospital records | 192 (32.1%) |
|  | Dyslipidaemia | Total cholesterol/HDL >7.0 \| Medication | 100 (16.7%) |
|  | Hypothyroidism | Thyroid-stimulating hormone >4 \| Medication | 20 (3.3%) |
|  | Hyperparathyroidism | Parathyroid hormone >6.7 | 85 (14.2%) |
| Mental health | Depression | Medical history | 172 (28.8%) |
| problems | Sleeping problems | Medical history \| Medication | 98 (16.4%) |
| Nervous system | Dizziness/Vertigo | Medical history | 13 (2.2%) |
| problems | Loss of consciousness | Medical history | 27 (4.5%) |
|  | Epilepsy | Medical history \| Medication | 16 (2.7%) |
|  | Encephalitis | Medical history | 24 (4.0%) |
|  | Peripheral neuropathy | Hospital records | 98 (16.4%) |
| Respiratory diseases | Asthma/Bronchitis/Chronic obstructive pulmonary disease (COPD) | Medical history | 186 (31.1%) |
|  | Pneumonia | Hospital records | 56 (9.4%) |
|  | Hay fever/Allergy | Medication | 21 (3.5%) |
| Hepatitis | Hepatitis B | HBV Surface Antigen >0 | 39 (6.5%) |
|  | Hepatitis C | HCV RNA >0 | 22 (3.7%) |
| Renal problem | Renal problem | eGFR <60 ml/min (MDRD estimation) | 28 (4.7%) |
| Cancer | Skin cancer | Medical history (validated in hospital/GP records) | 21 (3.5%) |
|  | Haematological cancer | Medical history (validated in hospital/GP records) | 7 (1.2%) |
|  | Solid organs cancer | Medical history (validated in hospital/GP records) | 12 (2.0%) |
| Blood conditions | Anaemia | Haemoglobin <8.5 (men) <7.5 (women) \| medication | 71 (11.9%) |
|  | Thrombocytopenia | Platelets <150 x 10^-9^ | 44 (7.4%) |
| Cardiovascular | Myocardial infarction (MI) | Medical history (validated in hospital/GP records) | 21 (3.5%) |
| Diseases (CVDs) | Angina pectoris | Medical history (validated in hospital/GP records) | 21 (3.5%) |
|  | Peripheral vascular disease (PVD) | Medical history (validated in hospital/GP records) | 13 (2.2%) |
|  | Hypertension | Systolic BP ≥140 \| Diastolic BP ≥ 90 \| Medication | 258 (43.1%) |
|  | Transient ischemic attack (TIA) | Medical history (validated in hospital/GP records) | 13 (2.2%) |
|  | Heart failure | Medical history (validated in hospital/GP records) | 6 (1.0%) |
| Bones and joint | Joint inflammation/Arthritis | Medical history | 50 (8.4%) |
| disorders | Osteopenia/Osteoporosis | DXA T-score <-1 \| Medication | 255 (42.6%) |
|  | Osteoporotic fracture | Medical history (validated in hospital/GP records) | 91 (15.2%) |
| Sexually transmitted | Chlamydia | Hospital records | 54 (9.0%) |
| diseases (STDs) | Gonorrhoea | Hospital records | 25 (4.2%) |
|  | Syphilis | Positive syphilis serology | 184 (30.8%) |
| Genitourinary | Urinary incontinence | Medical history | 30 (5.0%) |
| disorders | Erectile dysfunction | Medical history \| Medication | 87 (14.5%) |
|  | Liver problems | Medical history | 42 (7.0%) |
| Eye problem | Eye problem | Medical history | 108 (18.1%) |
| Vitamin D deficiency | Vitamin D deficiency | 25-hydroxy vitamin D <25 nmol/l \| Medication | 108 (18.1%) |
